# Branched-chain amino acids suppress the cumulative recurrence of hepatocellular carcinoma under conditions of insulin-resistance

**DOI:** 10.3892/or.2013.2497

**Published:** 2013-05-27

**Authors:** HITOSHI YOSHIJI, RYUICHI NOGUCHI, TADASHI NAMISAKI, KEI MORIYA, MITSUTERU KITADE, YOSUKE AIHARA, AKITOSHI DOUHARA, JUNICHI YAMAO, MASAO FUJIMOTO, MASAHISA TOYOHARA, AKIRA MITORO, MASAYOSHI SAWAI, MOTOYUKI YOSHIDA, CHIE MORIOKA, MASAKAZU UEJIMA, MASAHITO UEMURA, HIROSHI FUKUI

**Affiliations:** Third Department of Internal Medicine, Nara Medical University, Nara, Japan

**Keywords:** angiogenesis, BCAA, glucose, insulin, IR, HCC, VEGF

## Abstract

Branched-chain amino acids (BCAAs) reportedly inhibit the incidence of hepatocellular carcinoma (HCC) in patients with liver cirrhosis and obesity that is frequently associated with insulin resistance (IR). We previously reported that BCAAs exert a chemopreventive effect against HCC under IR conditions in rats. The aim of the present study was to examine the effect of BCAAs on the cumulative recurrence of HCC under IR conditions in the clinical practice. BCAA granules (Livact^®^, 12 g/day) were administered for 60 months following the local curative therapy for HCC, and several indices were determined. Treatment with BCAAs markedly inhibited the cumulative recurrence of HCC in patients with a high IR index [homeostasis model assessment (HOMA)-IR >2.5], but not in patients with HOMA-IR of ≤2.5. BCAA also improved the HOMA-IR, and the inhibitory effect was observed regardless of the serum albumin (Alb) levels. Similarly, BCAA treatment revealed a marked suppressive effect in patients with high fasting insulin [immune reactive insulin (IRI) >15 U/ml], but not with IRI of ≤15. BCAA treatment did not result in differences in HCC recurrence in patients with high and low glucose levels [fasting blood sugar (FBS) >110 and ≤110, respectively]. Furthermore, serum levels of the soluble form of vascular endothelial growth factor receptor 2 (sVEGFR2) were significantly inhibited along with these clinical effects. Our findings indicate that the inhibitory effect of BCAAs was achieved, at least partly, by coordinated effects of anti-angiogenesis and IR improvement. Since BCAAs are widely and safely used in clinical practice to treat patients with chronic liver diseases, BCAAs may represent a new strategy for secondary chemoprevention for HCC patients with IR. Moreover, our findings suggest that sVEGFR2 may be a useful clinical predictive marker for BCAA treatment under IR conditions.

## Introduction

Hepatocellular carcinoma (HCC) is now one of the major health problems worldwide. HCC is the sixth most common cause of cancer-related mortality. Patients with liver cirrhosis are at the highest risk of developing HCC (1). One of the reasons for the poor prognosis of HCC is its high rate of recurrence regardless of its etiology such as hepatitis virus C (HCV). Increasing evidence indicates that the high rate of recurrence, even after local curative therapy, is due to intrahepatic metastasis or multi-centric development of each respective neoplasm clone. Since the high-risk group of either primary or secondary HCC development appears to be clearer than the other types of tumors, it is likely that chemopreventive agents should be beneficial for improving the prognosis of HCC.

Any solid tumor including HCC that has not acquired its own new blood supply cannot grow to more than a few millimeters in size (2,3). Several studies have shown that neovascularization and angiogenic factors, such as vascular endothelial cell growth factor (VEGF), are significantly upregulated in human HCC samples (4–6). It has recently been reported that angiogenesis may be induced at the early stages of tumor formation and carcinogenesis (7–10). We previously demonstrated that angiogenesis was increased in a stepwise manner during the murine hepatocarcinogenesis process and that suppression of the VEGF signaling pathway markedly attenuated hepatocarcinogenesis (11). It has been reported that alterations of the hepatic microcirculation in the human liver occurs at an early stage of liver carcinogenesis in association with hepatic cellular changes or within the dysplastic nodules before the emergence of morphologically identifiable HCC (12).

Cross-sectional human studies have shown that insulin resistance (IR) (i.e., co-existence of high blood glucose and insulin levels) is a consistent finding in patients with type 2 diabetes mellitus (DM), and a close interaction between DM and HCC has been documented (13). DM is associated with a 2- to 3-fold increase in the risk of HCC regardless of the etiology of chronic liver disease (14,15). Experimental evidence for the contribution of HCV in the development of IR and DM has been found in an HCV-transgenic mouse model (16). Moreover, several human studies have demonstrated that IR is an HCC risk factor in patients with chronic hepatitis C (CHC) (17) and a similar close interaction was observed in patients with other morbidities, such as non-alcoholic fatty liver diseases (NAFLD) (18).

The branched-chain amino acids (BCAAs) are comprised of three essential amino acids: namely, leucine, iso-leucine and valine. Recent studies have shown that BCAA granules improved the nutritional status (e.g. hypoalbuminemia) and event-free survival in patients with liver cirrhosis (19,20). Furthermore, BCAAs were found to suppress the incidence of hepatocarcinogenesis in patients with HCV-related cirrhosis and obesity that is often associated with IR (21). BCAAs are also known to modulate insulin signaling. They induce glucose uptake and improve glucose metabolism in rats with liver cirrhosis (22,23). They also improve IR in patients with HCV-related liver diseases (24,25). Previously, we reported that IR itself significantly augmented VEGF-mediated hepatic neovascularization (26). We reported that BCAAs exert a chemopreventive effect against HCC along with suppression of VEGF expression and hepatic neovascularization in obese diabetic rats with IR (27). In contrast to the animal studies, single BCAA treatment failed to suppress the first and second recurrence of HCC after local curative therapy in clinical practice (28). However, this study was focused on the whole recurrence rate of HCC in patients with low albuminemia [serum albumin (Alb) level of <3.5] and not with or without IR.

In the present study, we examined whether or not BCAAs suppress disease recurrence in patients with HCC under the IR condition after they had received local curative therapy and the possible mechanisms involved.

## Materials and methods

### Patients and treatment

This study was conducted on 93 patients with HCC who were admitted to our hospital for treatment against HCC. Our first and second endpoint consisted of the effect of BCAAs on cumulative recurrence and overall survival, respectively. The number of patients in each group was based on previous studies. We previously reported that the combination of vitamin K (VK) and angiotensin-converting enzyme inhibitor (ACE-I) ameliorated the cumulative recurrence of HCC in 25 patients in each group (29). Similarly, another report showed the role of VK in the development of HCC in 25 patients in each group (17). We referred to these studies to determine the suitable number of patients in the present study. HCC was diagnosed with a combination of several imaging modalities: namely, ultrasonography (US), computer tomography (CT), magnetic resonance imaging (MRI) and hepatic angiography. The diagnosis of HCC was based on the 2005 diagnostic algorithm by the American Association for the Study of Liver Diseases (30,31). All lesions were hypervascular. By CT scan analysis, hyperattenuation was observed during the arterial phase in the entire portion of the tumor and hypoattenuation in the portal-venous phase. Since we did not encounter any unusual lesions that required needle biopsy for histological confirmation, we did not have any histological proof of the diagnosis of our patients. All patients received local curative therapy with percutaneous radiofrequency ablation (RFA) for prior HCC and were confirmed free of any residual HCC by several imaging modalities. The IR index was calculated on the basis of fasting values for glycemia and insulinemia according to the homeostasis model assessment (HOMA)-IR method as described previously (32). The clinical profiles, laboratory data and characteristics of the patients are shown in Table I. We recommended all patients to strictly stop alcohol intake. Until the end of the follow-up for recurrence, no patients received any additional therapy for HCC, such as interferon (IFN). All patients provided written informed consent before participating in this study. The study protocol was approved by the Institutional Review Board (IRB) of Nara Medical University (NMU06-003), and the study was conducted in conformance with ethical and humane principles, as well as the Helsinki declaration. The patients were randomly (using sealed envelopes) divided into either the control group or the BCAA-treated group. No placebo was used in the control group. The patients in the BCAA-treated group were given oral BCAA granules (Livact^®^, 12 g/day) continuously for 60 months, according to standard clinical practice. We assessed the BCAA adherence by questionnaire at each visit to the hospital. Since the adherence rate was more than 80% in all patients, we included all patients for analysis. We also divided the control patients into two subgroups according to HOMA-IR, immune reactive insulin (IRI) and fasting blood sugar (FBS), which were identical to the BCAA-treated groups.

### Study design

In the present study, we employed therapeutic modalities according to the algorithm of HCC treatment by the Liver Cancer Study Group of Japan (LCSGJ). In this algorithm, the therapeutic strategy is chosen based on the degree of liver damage as determined by LCSGJ and the characteristics of the tumor itself. The indications of RFA of LCSGJ are: i) three or fewer tumors measuring 3 cm or less, or ii) a solitary tumor with a major axis of 5 cm or less. In our university, an inter-departmental conference is held every week. We discuss the selection of therapeutic modalities with surgeons and radiologists for each HCC case. When RFA is selected in this conference, the patient is admitted to our department. After RFA, follow-up was conducted using enhanced CT and US (CECT and CEUS, respectively) once a month for the first three months. Regarding the safety margin, we set a 5-mm safety margin for all sides of the tumor for RFA. After one week, we confirmed the therapeutic effect by CEUS. If any viable HCC was detected during this period, we excluded it from the present study as non-local curative ablation by RFA. In the present study, we did not divide the patients by recurrent lesions. After the first three monthly observations, we included the HCC recurrence rate even when the recurrence occurred in the same lesion as the previous treatment.

To identify the recurrent HCC nodules, imaging studies such as US and CT were performed every 3 months. The serum tumor markers, namely, α-fetoprotein (AFP) and des-γ-carboxyprothrombin (PIVKA-II), were measured every 2 months using routine laboratory methods. Prior to and at 12 months after starting drug administration, alterations in angiogenic factors were evaluated using a TranSignal Angiogenesis Antibody Array (Panomics, Inc., Redwood City, CA, USA) in serum according to the manufacturer’s manual after the equalization of the protein content. We also examined alterations in VEGF (33), sVEGFR1 and sVEGFR2 in serum using an enzyme-linked immunosorbent assay (ELISA) kit (R&D Systems) according to the manufacturer’s instructions as described previously (11). When any recurrence was detected, the patient was excluded from the protocol and immediately treated for secondary HCC according to the LCSGJ algorithm. In the current study, all recurrent cases met the aforementioned RFA criteria. Accordingly, all patients with first recurrence were treated with RFA. Nevertheless, several patients with secondary recurrence received transarterial chemoembolization (TACE) and/or arterial infusion chemotherapy afterward. Liver transplantation is uncommon in Japan for patients with recurrent HCC.

### Statistical analysis

The variables of the characteristics of the enrolled patients were analyzed by the Mann-Whitney U test and the Fisher exact probability test. The cumulative recurrence of HCC was plotted using the Kaplan-Meier method, and the differences in the recurrence curves were tested using the log-rank test.

## Results

### Patient characteristics

The characteristics of the enrolled patients are documented in Table I. There were no significant differences among the patients of all groups in regards to age, gender, etiology of the disease and background liver function (Child-Pugh score). In addition, the tumor baseline, such as stage, number of tumors, AFP and PIVKA-II levels did not differ among the groups. BCAAs are widely used without serious adverse effects, and the safety of their long-term administration has been proven in clinical practice. In the present study, there were no severe toxic effects in any groups, and no abnormal laboratory data were found that could likely be related to the BCAA treatment. Accordingly, after randomization, we followed up all enrolled patients in each group until the detection of recurrence. We analyzed the demographic characteristics of all subgroups including HOMA-IR, IRI and FBS. There were no statistically significant differences. We also checked the unintentional increase in γ-GTP and the alteration in marked body weight. We observed that there was no significant difference among all groups.

### HCC recurrence

**Figure 1:**
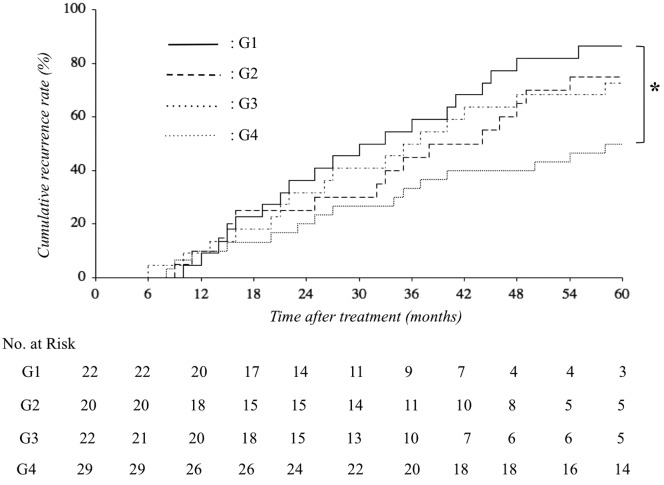
Effect of branched-chain amino acids (BCAAs) on the cumulative recurrence of secondary hepatocellular carcinoma (HCC) after local curative therapy for 60 months in the patients with high and low homeostasis model assessment (HOMA)-insulin resistance (IR). The BCCA treatment significantly suppressed the cumulative recurrence of HCC in patients with high HOMA-IR (HOMA-IR >2.5) when compared to the control patient group with HOMA-IR >2.5. On the other hand, no suppressive effect was observed in the patients with HOMA-IR ≤2.5 when compared to the control patient group with HOMA-IR ≤2.5. G1 and G2: control patient groups with HOMA-IR >2.5 and HOMA-IR≤2.5, respectively; G3 and G4: BCAA-treated patient groups with HOMA-IR ≤2.5 and HOMA-IR >2.5, respectively. Statistically significant differences between the indicated groups (^*^P<0.01).

**Figure 2:**
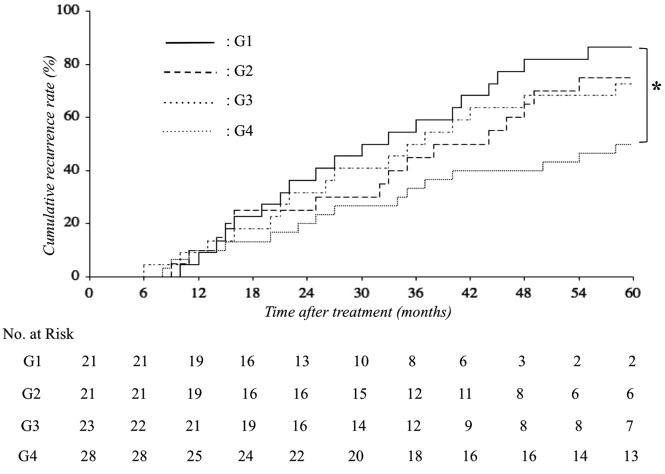
Effect of branched-chain amino acids (BCAAs) on the cumulative recurrence of secondary hepatocellular carcinoma (HCC) after local curative therapy for 60 months in patients with high and low IRI. BCAA treatment had a marked suppressive effect on the patients with high fasting insulin [immune reactive insulin (IRI) >15 U/ml), but not on the patients with IRI ≤15 when compared to the respective control group. G1 and G2, control patient groups with IRI >15 and IRI ≤15, respectively; G3 and G4, BCAA-treated patient groups with IRI ≤15 and IRI >15, respectively. Statistically significant differences between the indicated groups (^*^P<0.01).

**Figure 3:**
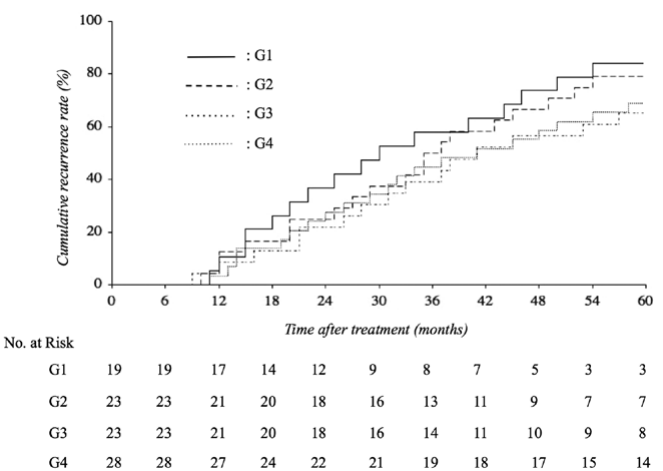
Effect of branched-chain amino acids (BCAAs) on the cumulative recurrence of secondary hepatocellular carcinoma (HCC) after local curative therapy for 60 months in patients with high and low glucose levels. The BCAA treatment did not result in any difference in HCC recurrence between patients with high and low glucose levels [fasting blood sugar (FBS) >110 and ≤110, respectively] when compared to the respective control group. G1 and G2, control patient groups with FBS >110 and ≤110, respectively; G3 and G4, BCAA-treated patient groups with FBS ≤110 and >110, respectively.

**Figure 4:**
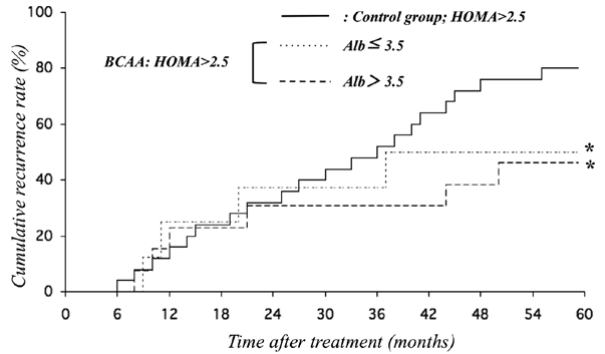
Cumulative recurrence of secondary hepatocellular carcinoma (HCC) after local curative therapy following treatment of the branched-chain amino acids (BCAAs) under conditions of insulin resistance (IR) [homeostasis model assessment (HOMA)-IR >2.5] for 60 months. BCAAs had a significantly suppressive effect on HCC recurrence regardless of the serum albumin (Alb) level (serum Alb >3.5 and ≤3.5) when compared to the respective control group. Statistically significant differences between the indicated groups (^*^P<0.01).

The results of the BCAA treatment are shown in Fig. 1. The BCAA treatment markedly inhibited the cumulative recurrence of HCC in patients with high IR index (HOMA-IR >2.5) as compared with the control group of patients with HOMA-IR >2.5. On the other hand, no suppressive effect was observed in the patients with HOMA-IR ≤2.5. Similarly, BCAA treatment had a marked suppressive effect in the patients with high fasting insulin (IRI >15 U/ml), but not in patients with IRI ≤15 as compared with the respective control group (Fig. 2). We observed that there were no significant differences in the alanine transaminase (ALT) levels between the control and BCAA-treated groups, indicating that the suppressive effect of BCAAs on the cumulative recurrence was not due to cytoprotective activity. Although the primary end-point of the current study was the cumulative recurrence rates, we also examined whether or not the survival curves of the patients were altered during the follow-up period as a second endpoint. We found no statistical differences between both groups (data not shown). In contrast to those of HOMA-IR and IRI, BCAA treatment did not show any differences in HCC recurrence between patients with high and low glucose levels (FBS >110 and ≤110, respectively) when compared to the respective control group (Fig. 3). We next performed subanalysis to examine whether BCAA exerts different effects on Alb levels under the IR condition. As shown in Fig. 4, BCAAs showed a significant suppressive effect on HCC recurrence regardless of the Alb levels (both serum Alb >3.5 and ≤3.5). All groups exerted 5–8% treatment-site local recurrence. However, there were no significant differences in the recurrence rate in each group in the current analysis.

### Plasma levels of VEGF-related factors and IR

**Figure 5:**
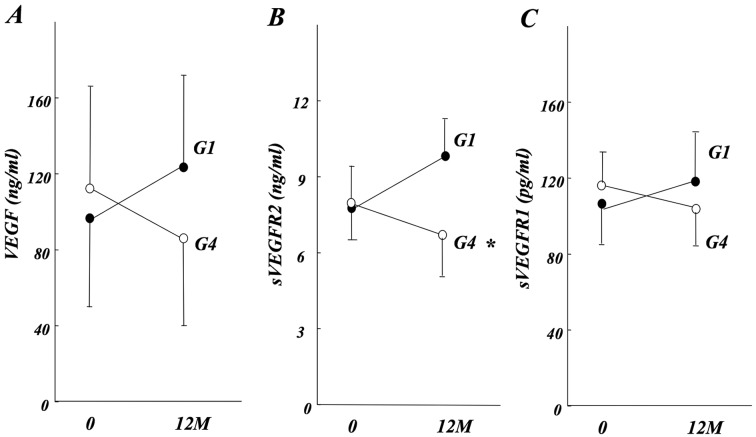
Effects of branched-chain amino acid (BCAA) treatment on the serum vascular endothelial growth factor (VEGF), sVEGFR1 and sVEGFR2 levels in the patients with HOMA-IR >2.5. (A) The serum VEGF level in the control group (G1) increased after 12 months whereas BCAA treatment (G4) markedly attenuated the VEGF level when compared to the pretreatment level, although it was not significant. (B) sVEGFR2 was markedly decreased by treatment with BCAAs whereas the serum level of sVEGFR2 in the control group increased. (C) There were no significant differences in sVEGFR1 between the pretreatment and the post-treatment levels in both groups. The data represent means ± SD (n=15). Statistically significant differences between the indicated groups (^*^P<0.01).

Since VEGF expression is predominantly altered by BCAA treatment (34), we measured the expression levels of VEGF-related molecules by ELISA in all patients. The serum VEGF level in the control group increased after 12 months whereas following BCAA treatment, the patients with high HOMA-IR attenuated VEGF level when compared to the pretreatment level, although it was not significant (Fig. 5A). We also elucidated the expression levels of the soluble forms of VEGF receptor R1 and R2 (sVEGFR1 and sVEGFR2, respectively). As shown in Fig. 5B, sVEGFR2 was markedly decreased by BCAA treatment whereas the serum level of sVEGFR2 in the control group increased. In contrast, there were no significant differences in sVEGFR1 between the pretreatment and post-treatment levels in both groups (Fig. 5C).

**Figure 6:**
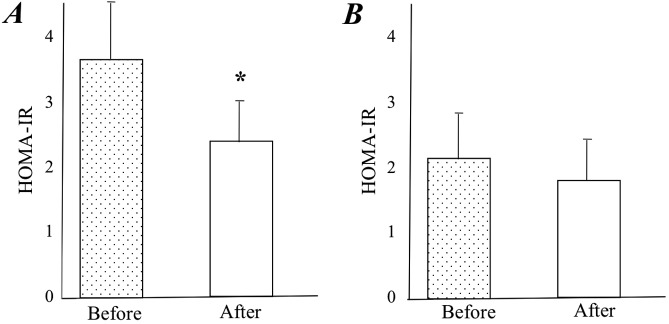
Effects of treatment with the branched-chain amino acids (BCAAs) on the insulin resistance (IR) status in patients with high and low HOMA-IR. The BCAA treatment for 12 months significantly decreased the median HOMA-IR score in the patients with high HOMA-IR although no marked difference was observed in the patients with low HOMA-IR (A and B, respectively). Statistically significant differences between the indicated groups (^*^P<0.01).

We also examined the alteration of the IR status by the HOMA-IR score. As shown in Fig. 6A, the treatment with BCAAs for 12 months significantly decreased the median HOMA-IR score in the patients with high HOMA-IR, although no marked differences were observed in patients with low HOMA-IR.

## Discussion

In the present study, we found that treatment with the clinically used BCAA formula markedly inhibited the cumulative HCC recurrence after local curative therapy under the condition of high HOMA-IR along with suppression of several indices. It is well-known that the IR status in patients with chronic liver diseases is usually associated with both high insulin and glucose levels (35). We previously reported that the IR status itself significantly augmented hepatocarcinogenesis (26) and BCAAs could suppress the development of pre-neoplastic lesions under the IR condition in rats (27). Accordingly, in the present study, we observed that BCAAs suppressed the recurrence of HCC in the patients with high IRI while glucose levels were not influence by the effect of BCAA treatment. A recent report showed that BCAAs suppressed insulin-induced hepatic tumor cell proliferation (36). We observed that IR was significantly improved by the BCAA treatment. This effect is also likely to play a clinically beneficial role since insulin is now recognized as a multi-functional protein involved in tumor cell proliferation and anti-apoptotic activity (26). These results indicate that the suppressive effect of BCAAs was, at least partly, mediated by the inhibition of insulin-induced HCC progression rather than glucose-induced effects.

BCAAs are known to exert multiple pharmacological activities (37,38). We observed that the inhibitory effect of BCAAs was correlated with a decrease in the angiogenic factor. We previously reported that BCAAs suppress VEGF expression during hepatocarcinogenesis in rats under the IR condition (27,39). IR itself has been shown to augment neovascularization (26). Recently, it has also been reported that BCAAs inhibit insulin-induced expression of VEGF by decreasing the stability of insulin-induced VEGF mRNA (40). We previously reported that VEGF and VEGFR2 interaction plays a pivotal role in HCC growth and hepatocarcinogenesis (11,41). It has been reported that the circulating sVEGFR2 level may provide insight into VEGFR2 activation (42). In renal cell carcinoma, the sVEGFR2 levels were significantly decreased by treatment with sunitinib, which is a multi-kinase inhibitor, including VEGFR, during the treatment cycle 1 (43). Moreover, the levels tended to return to near baseline after a 2-week treatment, indicating that sVEGFR2 was possibly a sensitive marker of anti-angiogenic therapy. These results indicate that BCAAs suppress VEGF and VEGFR2 interaction, and sVEGFR2 may be utilized as a useful clinical predictive marker for BCAA treatment under the IR condition. sVEGFR1 is the product of alternative mRNA splicing and is composed of only 6 of 7 immunoglobulin-like domains. sVEGFR1 is also reportedly a potential surrogate marker for disease progression in several types of cancers such as breast cancer and renal cell carcinoma. In HCC, it has been found that sVEGFR1 is correlated with poor prognosis although it is not an independent prognostic factor for HCC (44). In the present study, we did not find any significant correlation between sVEGFR1 alteration and HCC recurrence.

Although the primary end-point of the present study was the cumulative recurrence rates, we also examined whether or not the survival curves of the patients were altered during the follow-up period. No statistical differences were found among the groups (data not shown). Since the follow-up period in the present study was probably not long enough, no statistical differences were detected among the groups. Further long-term and large-scale studies are also required to verify whether or not the suppressive effect of BCAA on the cumulative recurrence will improve prognosis.

In addition, the inhibitory effect of single treatment of BCAAs seemed contradictory to our previous results (34). We previously reported that the single treatment of BCAAs could not exert enough inhibitory effect on HCC recurrence. The exact reason for this discrepancy is not clear at this time, however, the definition of IR may be one of the reasons. In the previous study, we defined IR with high insulinogenic-index by 75 g oral glucose tolerance test as well as HOMA-IR. The mechanism of IR in patients with chronic liver disease is known to be complicated (35). It has also been reported that BCAAs exert favorable effects on IR in several organs such as the liver, skeletal muscles and the adipose tissues (37,38). It is possible that the reaction of each tissue regarding IR improvement following BCAA treatment was different from the previous report. Further studies are required to elucidate the exact mechanism using large-scale clinical trials.

In conclusion, we showed that BCAA treatment significantly attenuates the cumulative recurrence of HCC for 60 months after local curative therapy under the IR condition along with suppression of several indices, such as VEGF, sVEGFR2 and HOMA-IR. These results indicate that BCAAs may represent a new anti-angiogenic strategy for secondary chemoprevention against HCC since BCAAs are widely used in clinical practice without serious side effects. Moreover, sVEGFR2 is a useful clinical predictive marker for BCAA treatment against HCC recurrence under IR conditions.

## Figures and Tables

**Table I:** Demographic characteristics of the enrolled patients.

Characteristics	Untreated control	BCAA	P-value
No. of patients	42	51	
Age^a^ (years)	62.2±14.8	63.6±15.3	0.408^b^
Gender, n			0.752^c^
Male	25	32	
Female	17	19	
Etiology, n			0.551^c^
HCV	32	36	
HBV	6	9	
Other	4	6	
Alcohol intake (g/day), n			0.648^c^
<40	33	42	
≥40	9	9	
Tumor stage, n			0.758^c^
I	25	29	
II	16	20	
III	1	2	
Tumor size^a^ (mm)	21.5±9.2	22.3±8.9	0.409^b^
No. of tumors^a^	1.74±1.18	1.84±0.96	0.082^b^
AFP^a^ (ng/ml)	81.8±176.5	84.4±186.8	0.349^b^
PIVKA-II^a^ (mAU/ml)	70.1±72.3	68.6±70.9	0.444^b^
ALT^a^ (IU/l)	70.3±34.3	72.2±34.9	0.449^b^
Alb^a^ (g/dl)	3.61±1.11	3.57±1.20	0.298^b^
HOMA-IR^a^	2.64±2.56	3.20±2.88	0.212^b^
IRI^a^ (U/ml)	12.34±7.33	14.58.±12.42	0.754^b^
FBS^a^ (mg/dl)	101.4±27.2	97.8±31.2	0.176^b^
Child-Pugh, n			0.829^c^
A	33	41	
B	9	10	

a Values represent means ± SD.

b,c Statistical analysis was performed with Mann-Whitney U test and Fisher exact probability test, respectively.

HCV, hepatitis virus C; AFP, α-fetoprotein; PIVKA-II, des-γ-carboxyprothrombin; ALT, alanine transaminase; Alb, serum albumin; HOMA, homeostasis model assessment; IRI, immune reactive insulin; FBS, fasting blood sugar.

## Abbreviations

AFP: α-fetoprotein
BCAA: branched-chain amino acid
EC: endothelial cells
FBS: fasting blood sugar
HOMA-IR: homeostasis model assessment-IR
IRI: immune reactive insulin
HCC: hepatocellular carcinoma
IR: insulin resistance
RFA: percutaneous radiofrequency ablation
VEGF: vascular endothelial growth factor
